# Supplementary material for: miRNA Mediated Regulation and Interaction between Plants and Pathogens
Source: Int J Mol Sci. 2021 Mar 13;22(6):2913. doi: 10.3390/ijms22062913 (PMC7999934; doi:10.3390/ijms22062913)
Supplement: Supplementary file 1 [file ijms-22-02913-s001.pdf]

Supplementary Table 1 Summary of regulation patterns of disease-related miRNAs in different plants

miRNA	Targets	Pathogens	Plant	References
**Positive regulation**				
miR863-3p	*ARLPK1,ARLPK2, SERRATE*	B: *Pseudomonas syringae*	*Arabidopsis thaliana*	[65]
miR393	F-box auxin receptors	B: *Pseudomonas syringae*	*Arabidopsis thaliana*	[31]
miR393b*	*MEMB12*	B: *Pseudomonas syringae*	*Arabidopsis thaliana*	[37]
miR393	auxin receptors	O: *Hyaloperonospora arabidopsidis*	*Arabidopsis thaliana*	[66]
miR161	*PPR*	O: *Phytophthora capsici*	*Arabidopsis thaliana*	[45]
osa-miR398b	*CDS1, CDS2, CCSD, SODX*	F: *Magnaporthe oryzae*	Rice (*Oryza sativa*)	[36]
osa-miR160	ARF TF：*ARF16*	F: *Magnaporthe oryzae*	Rice (*Oryza sativa*)	[67]
osa-miR7695	*OsNramp6*	F: *Magnaporthe oryzae*	Rice (*Oryza sativa*)	[68]
miR166k-166h	ethylene-insensitive 2 gene (*EIN2*)	F: *Magnaporthe oryzae*; *Fusarium fujikuroi*	Rice (*Oryza sativa*)	[69]
PN-2013	*TaMDHAR*	*Puccinia striiformis* f. sp. *tritici* (*Pst*)	Wheat (*Triticum aestivum*)	[70]
Can-miRn37a	ethylene response factors (ERF)	F: *Colletotrichum truncatum*	Chilli (*Capsicum annuum*)	[34]
ghr-miR477	*GhCBP60A*	F: *Verticillium dahliae*	Cotton (*Gossypium hirsutum*)	[71]
gh-miR159	*HiC-15*	F: *Verticillium dahliae*	Cotton (*Gossypium hirsutum*)	[43]
gh-miR166	*Clp-1*	F: *Verticillium dahliae*	Cotton (*Gossypium hirsutum*)	[43]
miR156	TFs	F: *Botryosphaeria dothidea*	*Populus trichocarpa*	[72]
miR1448	NBS-LRR	F: *B. dothidea*	*Populus trichocarpa*	[72]
miR164	NBS-LRR	F: *B. dothidea*	*Populus trichocarpa*	[72]
miR472a	NBS-LRR	F: *Cytospora chrysosperma*	*Populus alba* × *P. glandulosa* (84K)	[23]
miR172a/b	AP2/ERF	O: *Phytophthora*	Tomato (*Solanum*	[73]

		infestans	*lycopersicum*)	
**Negative regulation**				
miR396	GRF	F: *Plectosphaerella cucumerina; Botrytis cinerea; Fusarium oxysporum; Colletotrichum higginsianum*	*Arabidopsis thaliana*	[74]
ath-miR164c	*AtP5CS1*	B: *P. syringae*	*Arabidopsis thaliana*	[75]
miR164	*NAC4* (At5g07680)	*B: Alternaria brassicicola; Pseudomonas syringae;*flg22	*Arabidopsis thaliana*	[76]
miR394	*LEAF CURLING RESPONSIVENESS* (*LCR*)	F: *Botrytis cinerea*	*Arabidopsis thaliana*	[77]
miR393	auxin receptors	B: *Alternaria brassicicola*	*Arabidopsis thaliana*	[66]
miR398b	CSD1, CSD2	B: *Pseudomonas syringae pv tomato* DC3000	*Arabidopsis thaliana*	[78]
miR825*	TIR-NBS-LRR	F: *Botrytis cinerea*	*Arabidopsis thaliana*	[39]
miR5819	CPuORF3-*OsbZIP38*	F: *Magnaporthe oryzae*	Rice (*Oryza sativa*)	[79]
miRNA5075	CPuORF4-*OsbZIP27*	F: *Magnaporthe oryzae*	Rice (*Oryza sativa*)	[79]
miRNA2101	CPuORF7-SAM decarboxylase	F: *Magnaporthe oryzae*	Rice (*Oryza sativa)*	[79]
miR168	AGO1, AGO18	viruliferous (RSV and RDV-carrying) insects	Rice (*Oryza sativa*)	[80]
miR169	nuclear factor Y-A (NF-YA)	F: *Magnaporthe oryzae*	Rice (*Oryza sativa*)	[81]
miRNA528	L-ascorbate oxidase (AO)	rice stripe virus (RSV)	Rice (*Oryza sativa*)	[35]
miR159	*GAMYB*	O: *Phytophthora parasitica*	Rice (*Oryza sativa*) and Tobacco (*Nicotiana tabacum*)	[82]
nta-miR6019	NB-LRR	tobacco mosaic virus (TMV)	Tobacco (*Nicotiana tabacum*)	[27]

nta-miR6020	NB-LRR	tobacco mosaic virus (TMV)	Tobacco (*Nicotiana tabacum*)	[27]
tae-miR164	*TaNAC21/22*	F: *Puccinia striiformis* f. sp. *tritici* (*Pst*)	Wheat (*Triticum aestivum*)	[83]
miR408b	-	F: *Fusarium verticillioides*	Maize (Zea mays)	[84]
stu-miR482e	*NBS-LRR*	F: *Verticillium dahliae*	Potato (*Solanum tuberosum*)	[20]
miR482<br>miR2118	NBS-LRR	F: *Pseudomonas syringae*	Tomato (*Solanum lycopersicum*)	[5]
slmiR482e-3p	*NBS-LRR*	F: *Fusarium oxysporum*	Tomato (*Solanum lycopersicum*)	[85]
slmiR482f	NB-LRR	F: *F. oxysporum*	Tomato (*Solanum lycopersicum*)	[22]
slmiR5300	NB-LRR	F: *F. oxysporum*	Tomato (*Solanum lycopersicum*)	[21]
sly-miR6024	*I2* homologues	tomato yellow leaf curve virus (TYLCY)	Tomato (*Solanum lycopersicum*)	[86]
Md-miRLn11	*Md-NBS*	B: *Alternaria alternata* f.sp.mali	*Malus domestica* cv. *Golden Delicious*	[87]
miR472a	NBS-LRR	F: *Colletotrichum gloeosporioides*	*Populus alba* × *P. glandulosa* (84K)	[23]

B: bacterium; F: fungus; O: oomycete
